# Anatomy of an educational change: The safe learning model, Sierra Leone

**DOI:** 10.1007/s10833-022-09461-7

**Published:** 2022-08-12

**Authors:** Ciaran Sugrue, Elena Samonova, Daniel Capistrano, Dympna Devine, Seaneen Sloan, Jennifer Symonds, Aimee Smith

**Affiliations:** University College Dublin, Dublin 4, Dublin, Ireland

**Keywords:** Anatomy, Planned change, Change paradigms, Mixed-methods, policy analysis, reproduction, Transformation

## Abstract

This paper undertakes a critical analysis of a planned change, the Safe Learning Model (SLM), devised over time by Concern Worldwide, and implemented in 100 primary or elementary schools in a rural district of Sierra Leone. We situate the documentation pertaining to the SLM (micro) within its wider national (meso) and international (macro) context of influential policy texts. We undertake a mixed methods analysis of these macro, meso and micro documents, interrogated through the prism of various change paradigms (scientific management, progressivism, critical theory, teacher professionalism and social movement) and in doing establish where these various document clusters, their explicit and implicit influences, may be located along the arc of change paradigms, thus surfacing their ideological assumptions, intent and influences. The paper concludes that in seeking to improve the quality of teaching, learning, and living in this instance, scientific management casts long shadows. The power, perspectives and financial influence of international agencies dominate change discourses whereby ‘learning crises’ require urgent responses in the form of testing and measuring that prevail over more expansive pedagogical capacity building. Consequently, perpetuating a ‘weighing the pig’ mindset downplays or ignores the ecology of teaching and learning, particularly the centrality of teachers, as professionals and role models, more likely to be compliant than transformative.

## Introduction

There is widespread agreement that:

Today, governments are increasingly engaged in forms of global educational exchange and policy‐making, through membership in such diverse institutions as the Organisation for Economic Cooperation and Development (OECD), the Group of 8 (G8), the World Bank, the European Union (EU), the World Trade Organization (WTO), and the Association of Southeast Asian Nations (ASEAN). (Mumby et al., 2016, p. 1).

Consequently, there is considerable potential for policy borrowing across national boundaries, while international agencies have increased opportunity to influence educational systems and practices by the exercise of ‘hard’ and ‘soft’ power (Nye, 2008).

Due to fiscal, political, and cultural considerations, particularly in low-income countries, asymmetrical relationships of influence are widespread. Consequently, in developing country contexts, it is necessary to get inside interventions—planned change—to identify underlying assumptions and internal dynamics to develop a more sophisticated and potentially transformative understanding of change processes, to bring about ‘change for the better’, building educational capacities in a sustainable manner. The planned change under scrutiny in this paper is the Safe Learning Model (SLM), an intervention that has evolved over time, including efforts to improve literacy levels, well-being, and gender equality, designed by Concern Worldwide (hereafter, Concern) and implemented in a rural district in Sierra Leone, extending to 100 elementary or primary schools and their communities (see below).

Research questions are:

What are the dominant discourses of macro and meso policy documents, and to what extent are these replicated, translated, or transformed in SLM documents (micro), when ‘read’ through the lens of various change paradigms?

What lessons may be learned from the paper’s analysis about the problems and possibilities of planned educational change in a globalised world that may contribute to a better education for all?

First, we provide a succinct account of the education system in Sierra Leone, recognising the importance of context in bringing about change. Second, we provide a brief account of the planned change (SLM) in rural Sierra Leone. Third, in a section on change paradigms we review the assumptions that underpin a variety of approaches to planned change from scientific management, through progressivism, critical theory, teacher professionalism to social movement. This review enables us to situate the SLM in a wider educational change literature beyond the immediacy of its implementation surroundings, while it also provides an analytical lens that becomes the prism through which aspects of the planned change are interrogated. Fourth, we describe the mixed methods approach adopted to critically analyse macro (international), meso (national) and micro (local intervention) policy documents to contextualise more broadly the SLM planned change. Section five provides a detailed analysis of policy influences and how they metamorphose because of being processed through the macro, meso, and micro filters of influence, their agencies, and actors. The purpose of our analysis is to identify the extent to which these processes provide evidence of a more transformative approach to educational change or one characterised more by prescriptive, top-down perspectives reliant on a compliant incrementalism rather than an empowering professionalism, or an emergent ‘social movement’ paradigm, characterised as ‘radical and widespread pedagogical change in the Global South’ in opposition to a ‘scientific management’ approach to educational change (Rincon-Gallardo, 2020, p. 467).

### Education system: Sierra Leone

Sierra Leone became politically independent in 1961 after 160 years of British colonial rule. During colonial times, Sierra Leone was regarded as an educational centre in West Africa. It had the first secondary schools in the region (CMS Grammar School) and established one of the oldest universities in sub-Saharan Africa (SSA) (Fourah Bay College). However, mass schooling and universal education only appeared as an official political aspiration a few years before independence (Pai, 2013). According to the earliest data available, from 1971, Sierra Leone had a Gross Enrolment Ratio (GER) in primary school of 35% (UNESCO, 2020). By 1991, when the decade of civil war began, this ratio had increased to 43% (Ibid.). National statistics were not available for eight years after the beginning of the civil war, but estimates show a severe impact on children and the national education system. A survey conducted by the National Commission for Reconstruction, Resettlement and Rehabilitation (NCRRR) in 1998 revealed a generalised destruction of school buildings, including more than 2,000 primary and secondary schools (Government of Sierra Leone, 2001).

After the civil war (2002), the educational system experienced rapid expansion. In 2018, Sierra Leone registered a 112% GER, indicating that there is currently more enrolment in primary school than population of primary school age. This peak in demand has created enormous pressure on the system. In 2011, there were 52 pupils to every teacher registered on the government payroll. This pupil–teacher ratio varies from 48 in government schools to 129 pupils per government registered teacher in community schools; these community schools are not recognised by government, which are largely funded by communities, thus a major drain on limited resources (UNESCO, 2013). Public investment is one of the lowest in the world, with the government spending 4.8% of the GDP per capita on each student in primary school (UNESCO, 2020). Most schools in rural areas suffer from serious infrastructural inadequacies and lack of funding. The number of unqualified teachers in schools is reported to be 46% (UNESCO, 2020); in the 100 schools in the SLM study, the proportion of unqualified or ‘community’ teachers is 55%. This is the systemic context in which individual elements of the SLM planned change have been developed over time and put together to form a ‘model’ with a view to increasing impact on educational change, with particular emphasis on literacy and well-being.

### Safe learning model: its constituent elements and primary purposes

The SLM, implemented between 2017 and 2021, aimed to improve access to quality education in a safe learning environment; improve adolescent sexual and reproductive healt;, and enhance livelihoods, protection, and well-being of the children living in target communities. It is implemented in the Tonkolili district, located in the northern province of Sierra Leone, which is one of the poorest districts in the country. Most of the population in this district live in rural communities. Tonkolili has a literacy rate of 30% among adults (Margolis et al., 2016). Concern is an INGO that aims to eliminate extreme poverty.

The SLM’s working assumption is that children’s educational progress will be enhanced when they live in communities where there is more support for gender equality and children’s well-being. It includes efforts to improve children’s participation and outcomes in education through targeted and integrated interventions, including teacher professional development; measures to improve children’s literacy, social and emotional learning, and adolescent sexual and reproductive health (ASRH); and community decision-making and planning sessions to enhance gender equality in communities, as well as work with school management committees. To establish the efficacy of this model, a university-based research team has been commissioned by Concern to conduct a three-year evaluation of the SLM (2018–2021) in Sierra Leone. This mixed-methods research includes a randomised controlled trial and intensive qualitative immersion in a sub-sample of intervention communities.

### Change paradigms: values, assumptions, and possibilities?

Change, improvement, intervention, implementation, policy, along with educational reform and possibly transformation are terms frequently used interchangeably in change literature, doing nothing in the process to add clarity to the ‘messiness’ and ‘misery’ (McLaughlin, 2008) or what has earlier been labeled the ‘predictable failure’ of change efforts (Sarason, 1990). There appears therefore an inherent ambiguity and uncertainty regarding change processes, perhaps reminiscent of Heraclitus’ insight that: ‘No man ever steps in the same river twice, for it's not the same river and he's not the same man’. Despite change being a constant, Hargreaves (1994) makes the important point: ‘change is ubiquitous, development is optional’. From a planned change perspective in education, efforts to ‘tack the winds’ of change however are not a mere technical matter only, but are ‘concerned with emotional and spiritual engagement, social and moral justice, and improved performance in work and life’ (Hargreaves et al., 2014, p. 2).

Whether approaches to educational change are perceived as ‘subversive’ (Postman & Weingartner, 1975) or ‘conserving’ (Postman, 1979), the field has evolved over time through a variety of models or ‘paradigms’. For Kuhn (1970), a paradigm is ‘a world view’, a way of framing problems towards solutions. Taylor (1911) (often described as the ‘father of scientific management’, the dominant paradigm) pronounced its principles, placing particular emphasis on efficiency, though cooperation and developing full potential were also included, if subsequently neglected. Arising from the influence of scientific management, and other influences, behaviourism among them, by the 1920s ‘research in education had become more technical than liberal … more narrowly instrumental than genuinely investigatory in an open-ended playful way’ (Condliffe Langemann, 2000, p. 236). Educational change paradigms in the intervening century have struggled for position, presence, and influence against the dominance of ‘scientific management’ in its various guises, the more recent to emerge being ‘social movement’ (Rincon-Gallardo, 2020). Each paradigm has its own assumptions and logics, strengths, and limitations, while the boundaries between alternatives to scientific management are more porous, perhaps more accurately described as dispositions or orientations rather than paradigms. Nonetheless, Table 1 summarises their dominant characteristics; subsequently, we describe and critique this rainbow or arc of intentions regarding educational change efforts.

**Table 1:** Educational change paradigms: characteristics and keywords

Characteristics	Scientific management	Progressivism	Critical theory	Teacher professionalism	Social movement
Leadership	Control/ Compliance/ hierarchical	Liberal individualistic	Transformative/ radical	Distributed/ collaborative	Autonomy/creativity networked, distributed
Core values	Achievement/ efficiency	Child-centred Engagement curricular flexibility	Praxis Hidden curriculum conscientisation	Autonomy professional capacity-expertise	Learning efficacy
Core practices	Prescription mandates External accountability	Learner-led flexibility active engagement of learners	Deliberation awareness raising solidarity	Emphasis on professional learning/ enhancing agency through collaboration/ connection	Dialogue deliberation Internal accountability
Relies on	External incentives resources	Teacher imagination & learner engagement; constructivist pedagogies	Agency resourcefulness networks critical consciousness	Professional responsibility/ learning/ autonomy	Intrinsic motivation resourcefulness
Stance on change	Stability incrementalism	Childhood ‘flourishing’ as preparation for a changing world	Bottom-up liberation	Transformative capacity building through collaborative endeavour	Cultural renewal radical innovation
Keywords (Orientation)	Standards Efficiency testing measurement accountability	Liberal child-centred process-oriented active-engagement constructivist	Ideology emancipatory liberation agency social justice	Teacher agency capacity building collaboration professional community	Collective action oriented grassroots solidarity strategic transformative

Table 1 builds on the work of Rincón-Gallardo (2020) by expanding the range of paradigms, as part of this paper’s analytical lens while also creating a mapping of the field as it has evolved over time. At the turn of the twentieth century, as education as a discipline was seeking to establish a firm foothold within the academy, at least in a US context, Thorndyke and his narrow behaviourism gained the upper hand over Dewey’s progressivism (Condliffe- Langemann, 2000). The former’s disposition, influenced by Taylorist thinking, created ‘an emphasis on testing and tracking in education and on test development’ (Ibid., p. 235). This dominant thread in the field of educational research, ‘the most recent manifestation of scientific management in education reform’, finds expression in ‘The triad of standards, testing and accountability’ (Rincon-Gallardo, 2020, p. 468). This paradigm has been invigorated in recent years by the emergence of ‘big data’ while globalisation and the efforts of international agencies such as the World Bank (WB) and the OECD have used their positions, power and resources to dominate and, in some instances, dictate a particular narrative of reform with the characteristics identified in Table 1. Nevertheless, despite its dominance, critics point to its persistent failure to bring about desired reforms (McLaughlin, 2008; Sarason, 1990).

By contrast, progressivism retains its optimism regarding ‘the innate goodness’ of the child, reflected in Rousseau’s Emile (1762) and subsequently reframed in various ways by reformers such as Froebel, Montessori, as well as Dewey and others, who sought, in various ways, to undermine fixed notions of intelligence, thus emphasising the importance of nurture rather than nature. Critics suggest that its attendant pedagogies favour the middle classes, building on existing socio-cultural capital in comparison with their working-class peers who may benefit more from greater direction, thus the necessity for some ‘stick’ rather than over-reliance on the carrot of intrinsic motivation (Sugrue, 1997; Hargreaves, 2020). Progressivism, to a significant extent, has assumed the role of an ‘alternative’ to the dominant scientific management paradigm while aspects of its pedagogies have been incorporated into the mainstream, (use of resources and learner’s active engagement) softening the harder edges of the former, while not fundamentally altering its dominant architecture. These various discourses have waxed and waned throughout the twentieth and into the twenty–first century. More recently, both paradigms are accused of being too lumbering in their response to the pace of change (Rincon-Gallardo, 2020). Nonetheless, although there is recognition also that no matter how potent teacher agency may be to bring about reforms at a local or classroom level, there continues to be major structural constraints (Datnow, 2020; Datnow & Park, 2019).

Critical theory has been to the fore in espousing more radical roots, putting major emphasis on actor agency at grassroots level, while seeking to undermine what it identifies as oppressive constraints on learners, teachers, and society. Inspired by the work of Freire (1970), this too has become a broad church, embracing different theoretical perspectives such as post-structuralism and post-colonialism (Fanon, 2008; Said, 1993), while assiduously seeking out and being critical of evidence of dominance and oppression (Apple, 2004; McLaren, 1993; McLaren & Kincheloe, 2007; Steinberg, 2009). And, while along with progressivism it seeks to position learners and learning centre stage, it is much more critical of context, curriculum and attendant systemic apparatus that perpetuate the status quo rather than expose its oppressive and coercive tendencies and tentacles. Rather like its progressive near relative, it has been consigned to an alternative slot, a minority interest, in the pantheon of change paradigms, arguably an important trope that affirms the dominance of a scientific paradigm.

By contrast, teacher professionalism and professional community, perhaps seeking common cause with progressives as well as aspects of the critical tradition, have enjoyed greater visibility in the rivers of reform. Nonetheless, influenced by globalisation, the accelerating pace of change, as well as the influence of ‘systems thinking’ (Senge, 1990), there is growing concern, fostered further by Covid-19, that ‘Tinkering towards Utopia’ (Tyack & Cuban, 1995) is no longer a complacent incrementalism educational change can afford. The fulcrum of change within this paradigm is teachers, working in community, continuously building their professional capacity riding the waves of change, while simultaneously systems seek particular reforms, thus a tussle between policy-makers and practitioners, as initiatives move from ‘statehouse to schoolhouse’. Consequently, Datnow (2020. p. 435) identified ‘*co-construction processes’* as a desirable ‘legitimate compromise’ (May, 1996), whereby, ‘a dynamic relationship between structure, culture, and agency was helpful in making sense of the complex complexities of school improvement’ and in the process was critical of ‘the technical-rational model of school reform in which the causal arrow of change moves only in one direction’ (Datnow, 2020, p. 435). Emerging therefore has been an increasing awareness that ‘teachers matter’ (OECD, 2005), their lives, and work in change efforts are indispensable (Day, 2012; Day & Gu, 2010; Day et al., 2007). Two of the most recognisable proponents of teacher professionalism acknowledge that given the recent experience of learners, parents, and teachers imposed by Covid restrictions, it will be necessary to build a more ‘*open professionalism*’ to ‘*build professional capital and community’* but this requires a delicate balance between systemic reform and teacher agency and community (italics original) (Hargreaves & Fullan, 2020). Nevertheless, one of these authors is also on record that education is far too important to be left to teachers only! (Fullan, 2003).

Such endeavours, even in very well-resourced educational systems have had, at best, mixed and partial success. What happens when these paradigms are ‘exported’ to less developed contexts, frequently by international NGOs and other international agencies such as WB, UNESCO, EU and national aid agencies?

International literature recognises that knowledge on how to effect change has remained rather elusive, stating:

Over the past several decades, many change efforts have moved rapidly across education but with little real knowledge as to how to effect the improvements envisioned by reform advocates (or even whether those improvements were possible). (Bryk, 2015, p. 468).

Lack of success is frequently explained by an underestimation of the complexity of the challenges. Rather, it is necessary to recognise that: ‘complexity is real, and it cannot be side-stepped by standardising all activity that endeavours to “teacher-proof” instructional environments’ (p. 473). Although working locally on available evidence has potential, which too depends on expertise, in developing country context all too frequently in relatively scarce supply, making an ‘improvement paradigm’ more challenging to execute. In a systematic review of the importance of teachers’ pedagogical repertoires, several SSA studies reveal: ‘programs that alter teacher pedagogy or classroom instructional techniques’ were significantly more influential than ‘all other types of programs combined’ (Conn, 2017, p. 863). Apart from establishing the critical importance of teacher pedagogies in this meta-analysis, the nature of the support necessary for teachers if their pedagogical routines are to be altered includes:

Training to teachers on strategies to integrate books into their lesson plans; the ‘intensive and systematic professional development’ included demonstration lessons by mentors, monthly coaching visits by staff, one-on-one reflections sessions after monitoring visits, and after-school workshops for both teachers and school administrators. (Conn, 2017, p. 880).

Coaching, not without reason, and as a significant dimension of a ‘teacher professionalism paradigm’ has become a prioritised practice for ‘donors, governments and implementers’ as they ‘have placed their faith in coaches’, but such ‘magical thinking’ is not without its challenges (Burns, 2020). So little is known about ‘the presence and scope of teacher coaching programs as they currently are being implemented across the U.S. or elsewhere around the world’ (Kraft et al., 2018, p. 576). However, coaching is unlikely to be successful if reform efforts persist in ‘trying to solve the problem that requires professional skills and expertise’ by prioritising ‘bureaucratic levers of requirements and regulations’, reflective of a scientific management mindset, one that teacher professionalism in particular seeks to redress (Mehta, 2013, p. 463).

While feeding programmes may improve cognitive functioning, textbooks and school requisites make a positive contribution to the learning environment, it is ‘only interventions in pedagogical methods have the potential to affect how students learn in addition to what they learn, and this “learning how to learn” process may prove to have long-term benefits for students’ (Conn, 2017, p. 889). The quality of teaching and learning is where ‘the rubber meets the road’, particularly in contexts where parents and adults are frequently not equipped to support school work due to their own literacy levels. Additionally, there is evidence that an ‘increase in teacher subject knowledge raises student performance’ but this may be true ‘only in countries at a higher stage of development’ than the poorest countries, such as Sierra Leone (Bietenbeck et al., 2018, p. 571). These authors conclude that ‘the low skills of teachers in developing countries may limit the impact of other educational interventions (for example, when it comes to using textbooks effectively). Hence, it seems essential to increase the skills of the teacher workforce’ (Ibid.)

It is in such contexts that evidence of an emergent paradigm, social movement, gives grounds for optimism; described by Rincon-Gallardo (2020, p. 472) as being ‘grounded on the philosophy and practice of progressive education and critical pedagogy’ yet ‘offers a theory of action to transform entire educational systems, not just individual schools’. He is particularly optimistic that educational change as social movement is not merely a theoretical nicety, but is already manifest in unlikely places, particularly the Global South, drawing on empirical evidence from Mexico, Colombia, parts of India as well as Egypt. Such social movements he attests serve ‘remote and historically marginalized communities’, arise from ‘grassroots efforts’, have potential to ‘access institutional power’ and thus ‘change relationship between central leadership and schools’ as well as improve ‘student outcomes’ (p. 473). Such bottom-up collective agency resonates with the view that ‘The capacity of a social movement for effective action depends largely on the depth, breadth, and quality of leadership able to turn opportunity to purpose’ while recognising that such efforts are fraught with risk and uncertainty, hardly the most promising of contexts for educational change (Ganz, 2011, p. 274).

A particular feature of these change efforts is the way ‘they are redefining how adults and young people interact in classrooms, and how policy and practice interact with each other (through horizontal relationships of dialogue and mutual influence, rather than vertical relationships of authority and control’ (Rincon-Gallardo, 2020, p. 474). Core elements of social movements are collective translation of values into action, commitment, persistence, solidarity, and evolving strategy in the face of structural constraints.

Each paradigm has strengths and limitations, while in more recent years through the influence of systems thinking bottom up as well as top down has gained legitimacy. Even at an international level, where INGOs engage with national policy-makers from a variety of contexts, there is emerging evidence that it is necessary to move beyond policy borrowing to what Datnow has identified above as ‘co-construction’ with potential to blend with aspects of ‘systems thinking’ (Mehta & Peterson, 2019). For such collaborative endeavours to lead to co-construction and aspects of systems thinking, trust through relationship building over time is critical, even among small elite groups. Such challenges are magnified at meso and micro levels when culture, language, knowledge and power relations are frequently amplified depending on the actors involved. While communities of practice hold out considerable promise, it is necessary also to be aware of power dynamics in such trans-national engagements. Whatever the change paradigm, there is increasing evidence and acceptance that ‘smarter teachers make smarter students’ (Hanushek et al., 2019, p. 858), reinforcing the view that ‘teachers matter’(OECD, 2005), thus paradigms that pay appropriate attention to building their capabilities are more likely to have sustainable impact on learners.

Within the arch of possibilities that these educational change paradigms suggest, where is the SLM planned change to be located, and critiqued in terms of its intent as it is contextualised within its wider macro and meso policy framing?

## Methods and data

This paper deploys a mixed-method analysis of SLM documentation while situating this planned change within wider policy rhetorics, seeking out the influences, continuities, and ruptures in and between texts from macro, meso and micro policy levels. This approach is considered apposite since it is widely recognised that global discourses, shared by international agencies and INGOs, influence decisions and practices at the national and the grassroot levels of educational change efforts. Analysis begins with selected macro level policies and subsequently identifies the extent to which these global rhetorics are reproduced, modified, amended, or transformed in documents (re-)produced at national (meso) and grassroots (micro) levels.

For this analysis, we selected a range of documents published by the main international actors that cooperate closely with the Sierra Leonean government and support the educational sector financially (macro), by the government of Sierra Leone itself (meso) as well as the documentation of the SLM model (micro). Macro policies analysed included those most recently published by the main donors in the educational sector (WB, UN, UK, USA, Ireland and GPE) as well as the recent educational strategy published by the African Union. The meso level documents comprise the most recent educational sector plan of the Sierra Leonean government along with the professional standards for teachers. Both document sets express mainstream views on the problems and solutions in education, reflecting global rhetoric on education reform (Fuller & Stevenson, 2019; Sahlberg, 2016). At the micro level, SLM documents include manuals, guides and curricula for clubs and conversation groups. Table 2 provides a summary of the documents used in our analysis.

**Table 2:** Documents analysed in each level

Dimension	Organisation/component	Number of documents
Macro	World Bank, United Nations, African Union, Global partnership for Education and UNICEF, UK Aid, Irish Aid, US Aid	7
Meso	Government of Sierra Leone, Sierra Leone Teaching and Service Commission	2
Micro	Safe Learning Model—Project Information Document Safe Learning Model – Literacy Manual Teacher Learning Circle Workbook Resource Manual for Learning Coaches SRGBV Teacher Training Social and Emotional Learning Curriculum School Club Curriculum Community Conversations Facilitation Guide Living Peace Groups Curriculum	9

**Fig. 1:**
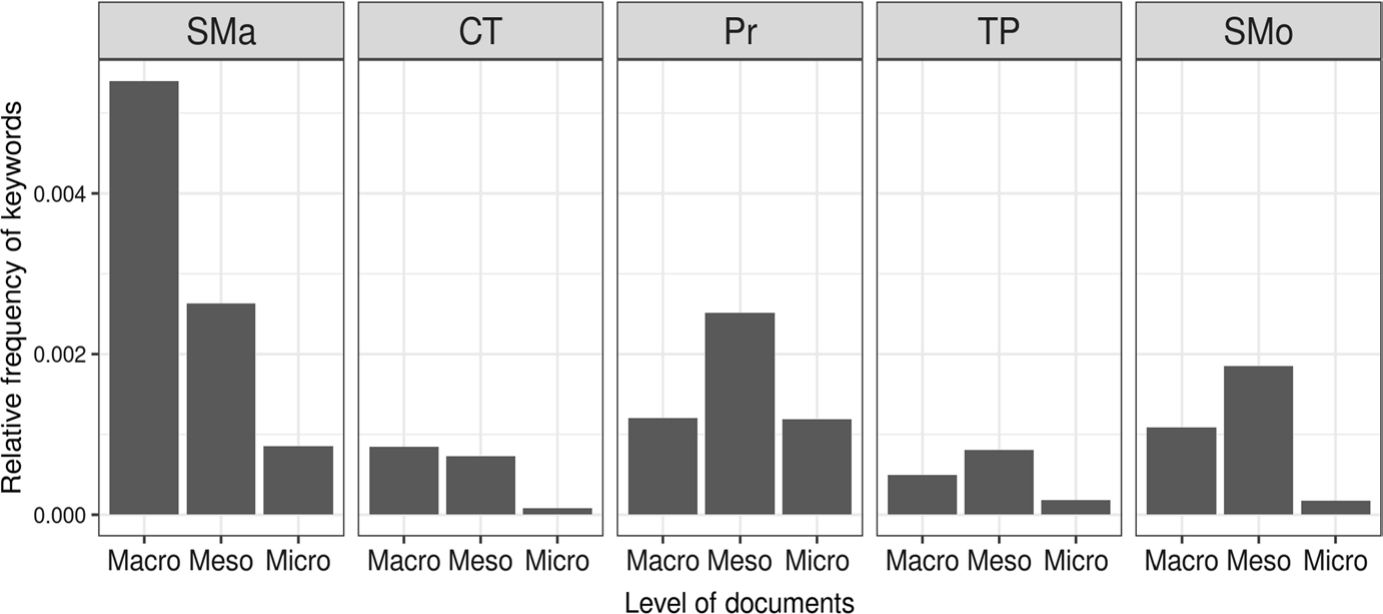
Relative frequency of key terms in macro, meso and micro document sets. Scientific Management (SMa), Critical Theory (CT). Progressivism (Pr). Teacher Professionalism (TP), Social Movements (SMo)

This mixed-methods analysis has two main components: initial quantitative textual analysis followed by qualitative interrogation employing elements of critical discourse analysis and grounded theory. Both quantitative and qualitative analysis employed abductive reasoning (Alvesson & Skoldberg, 2000). Initially, an exploratory analysis utilising the keywords attributed to each paradigm enabled us to identify the prevalence of change discourses across the three levels of documentation (Fig. 1). A quantitative approach was preferred in this exploratory phase for a systematic examination of the 18 documents. Each document was converted into a corpus and the relative frequency of key terms compared, identifying the prevalence of these terms across these documents. As part of this analysis, we have also measured the degree of lexical similarity between documents (Benoit et al, 2018) which provide empirical support for the grouping of documents into three categories: macro, meso and micro. As an initial foray into the three document sets, Fig. 1 indicates the prevalence of the five paradigms in each group of documents.

Initial quantitative textual analysis identified the prevalence of keywords in the documents, qualitative analysis focuses on their meanings. Qualitative textual analysis began with close reading of all 18 documents and initial inductive coding of the texts. Following the rules of circular deconstruction based on the methods of grounded theory, data were separated into meaningful segments, coded in open, axial and selection coding systems and then categorised to formulate the main conceptions related to the topics foregrounded in the quantitative analysis (Glaser, 1992). The codes that emerged from this process were approached from the perspective of how the dominance of the scientific management paradigm, evident in Fig. 1, was used to frame ‘the problem’. This metamorphosis of the problem and its solution is reported on in the empirical section of the paper.

### Analysis: anatomy of reform discourses–from crisis to intervention

**Fig. 2:**
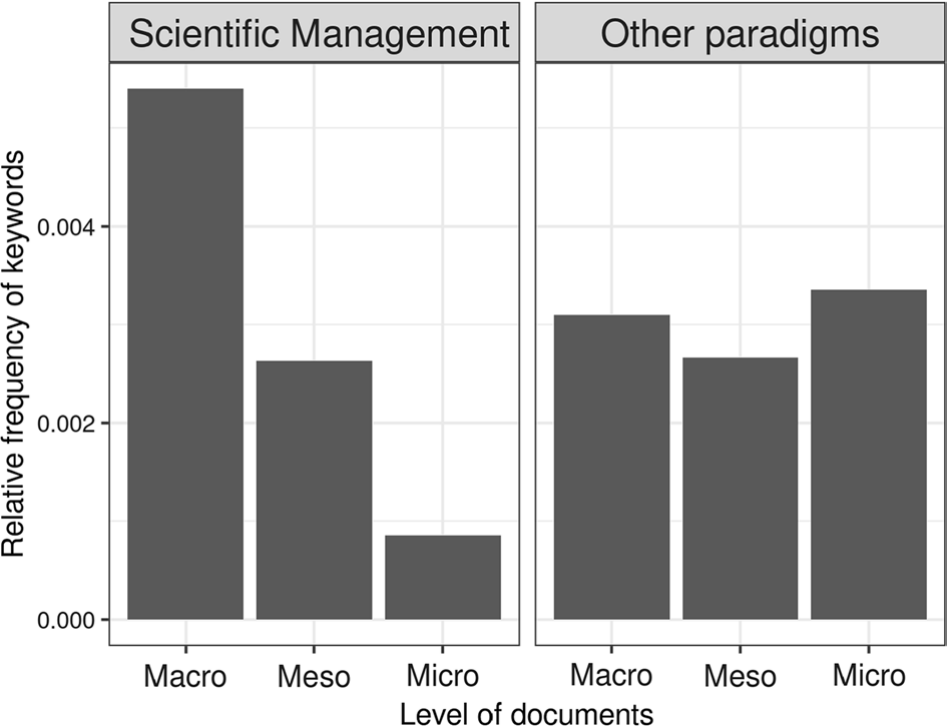
Relative frequency of keywords from the scientific mangement paradigm and other paradigms combined, across the documents. Scientific Management (SMa), Critical Theory (CT). Progressivism (Pr). Teacher Professionalism (TP), Social Movements (SMo)]

The dominant presence of scientific management paradigm language is particularly striking in macro documents,^1^ with relative absence of other paradigms also indicated. Consequently, at the risk of being reductionist, we conflated the keywords (Table 1) in the alternative paradigms to scientific management and repeated the analysis (Fig. 2).

**Fig. 3:**
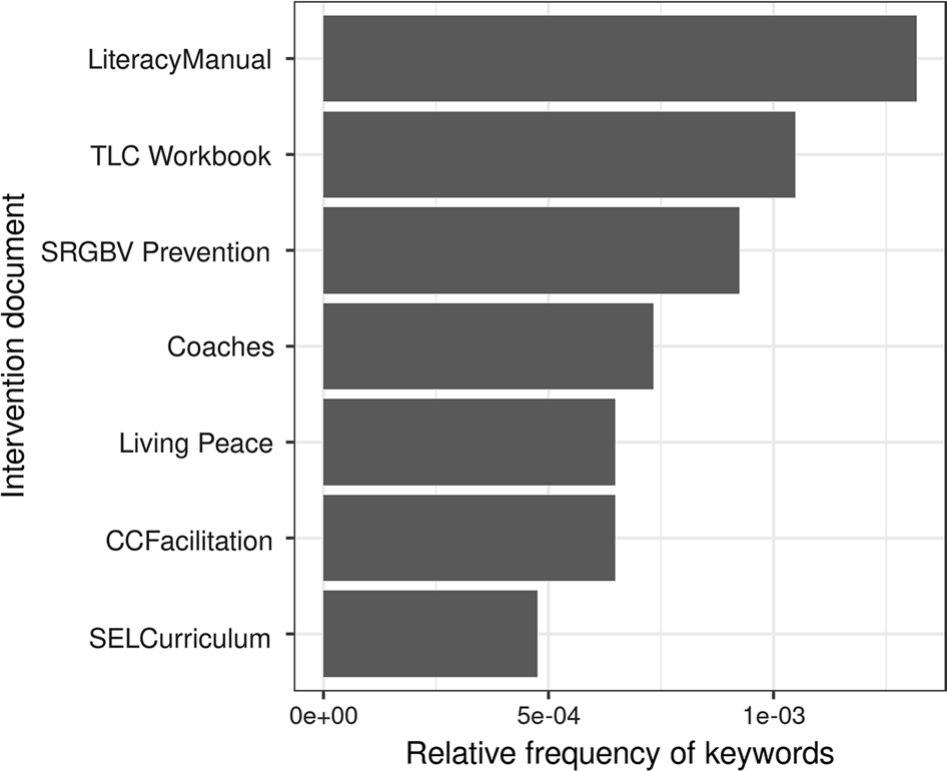
Relative frequency of keywords from the scientific management paradigm in each micro documents

The evidence above indicates the dominance of scientific management at macro level, while arguably this is softened somewhat by the increased presence of other paradigms at meso and micro levels. Beyond this relative paradigm ‘head count’, and mindful of Datnow’s evidence above regarding co-construction, Fig. 3 indicates the presence of scientific management terms in each of the micro documents, conscious of a bottom-up as well as top-down approach to bringing about change.

Even though these micro documents were written by actors other than those responsible for the macro and meso texts, the scientific management paradigm continued to have a strong presence, particularly though not surprisingly in relation to literacy. Consequently, we sought to combine quantitative and qualitative methods in our efforts to shed light on the framing and creation of the intervention. Given the dominance of the language of scientific management, it appropriates the terrain, framing the problem in its own image. Our immersion into macro and meso texts identified several central discourses deployed as tropes to frame the problem. Prevalent among them was crisis, measurement, assessment and tests. Dominating the discourse, not only provides the language in which the problem is framed, but the manner of its solution.

The concept of crisis has become fundamental to modern society (Cordero, 2017). Literature on development and education policy describes various kinds of crises: food, environment, migration, COVID 19 pandemic, economic, poverty and politics. As Gamble (2009) argues, this ‘crisis’ rhetoric justifies urgent programmes and quick decisions that provide interventions and strategies for action. Education is no exception; the language of crisis has been highly prominent in international and national documents for decades, while understandings of the nature of this crisis have changed during that time (Smith, 2016).

**Fig. 4:**
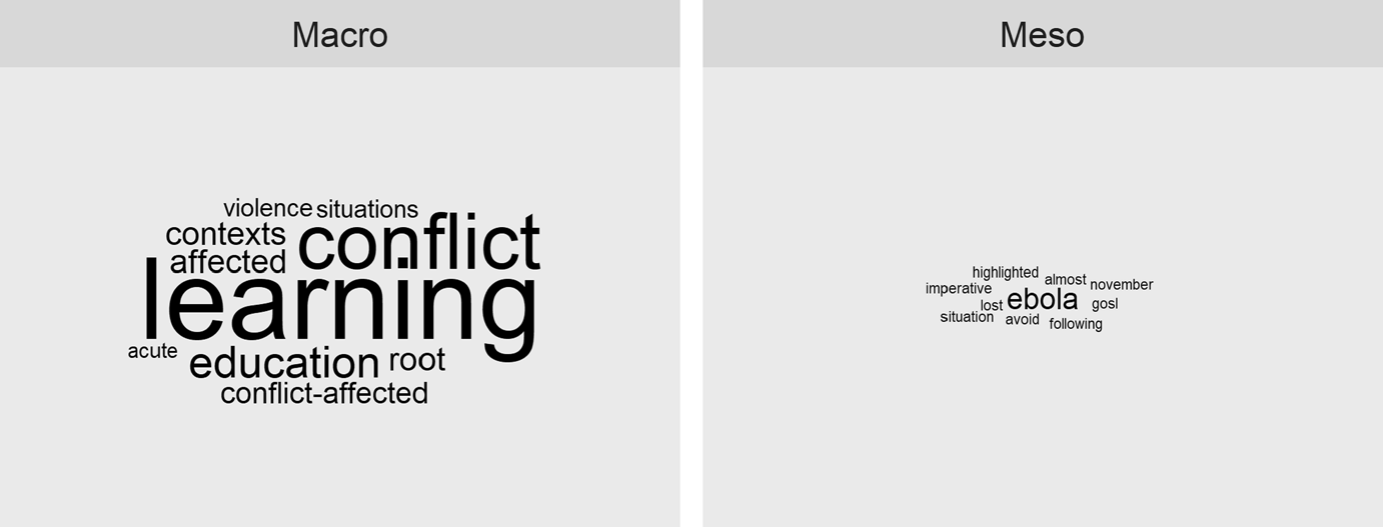
Ten most prevalent terms preceding or following the word “crisis”

Analysis of macro and meso documents reveals that the concept of crisis is widely used in these texts. For example, the education policy strategies of both UK DFID (2018) and USAID (2018), as well as the World Development Report (WB, 2018), are prominent in this regard. The term *crisis* is mentioned 28 times in the USAID document, 45 times by DFID, and 54 times in the WB report. Both the UKAID and WB documents contain sections entitled ‘learning crisis’. The UN’s Incheon declaration uses the term crisis 14 times, whereas the African Union document uses the term once. The prevalence of this term is lower among the national (meso) documents where ‘crisis’ is cited 11 times in the Government's Education Sector Plan and not mentioned in the Teaching and Service Commission policy. Finally, the term is absent from all the SLM documents (see Fig. 4).

Although the word is frequently used in the international documents, closer examination of texts indicates that this word may have different connotations. Figure 4 shows the most frequent words preceding the word ‘crisis’ which are learning, conflict, violence, education, and conflict-affected. Qualitative analysis reveals two prominent uses of crisis: (1) a broad variety of political, economic, and social failures and (2) failure of educational systems. The latter is often labelled as a ‘learning crisis’ in the documents. These connotations are not mutually exclusive. Rather, the learning crisis is frequently placed in the broader context of other crises such as poverty, gender inequality, and conflicts. Interestingly, development agencies tend to discuss the educational problems within the broader context of their developmental work on prevention and mitigation of other crises, while international agencies are more likely to focus explicitly on the learning crisis. For example, in its declaration of work priorities, DFID claims that it will ‘deliver for children whose education is disrupted by conflict, protracted crises and natural disasters’' (2018, p. 26). Similarly, although the WB acknowledges the problems of conflicts and other crises, it argues that they are only some of the issues that contribute to the overall ‘learning crisis’ resulting in low educational outcomes that should be addressed in the first instance.

While the language of crisis has been present for decades (Smith, 2016), the concept of the ‘learning crisis’ is relatively new. It appeared initially in a UNESCO (2013) report, ‘Global Learning Crisis’, claiming that, despite growing enrolment rates, ‘250 million children cannot read, write and count well, whether they have been to school or not’ (2013, p. 2). This report is significant for two reasons. First, it was the first sign of a shift from a more traditional focus on school enrolment to the quality education agenda. Second, it explicitly links the concept of quality education with learning outcomes in literacy and numeracy. Subsequently, this focus on the outcomes as quality markers becomes foundational to a significant shift, a new focus in education, resonant with a scientific management paradigm of educational change, its preoccupation with achievement expressed in numbers.

Our analysis indicates that the discourse of learning crisis as low educational outcomes is present in all international documents, though only indirectly in some. WB and DFID identify the current situation in education as a learning crisis, while other actors address the same set of problems and solutions without explicitly referencing this term. DFID (201, p.9) identifies the problem as ‘ensuring children learn the basics of literacy and numeracy’ as the means of ‘tackling the learning crisis at its root’.

Similarly, USAID (2018, p.7) wants to ‘improve learning outcomes’ by increasing ‘access to quality basic education for all’ especially ‘marginalized and vulnerable populations’. Through the ‘algorithms’ of these macro documents, there is consensus that learning outcomes need to be prioritised as quality markers of education. The consensus extends also to agreement on the perceived causes of such a crisis of learning outcomes. They include low school readiness, lack of skills among teachers, ineffective school management, lack of inputs (such as textbooks and school infrastructure) and bad governance practices including the lack of cooperation and unproductive spending (WB, 2018).

Preoccupation with learning outcomes as markers of quality in education has their genesis in neoliberal ideologies of education as human capital formation for the purposes of economic growth (McMahon & Okatch, 2013). Such ideological underpinnings are clearly visible in almost all international documents. USAID discusses the benefits of education thus:

Human capital development through education contributes to addressing poverty reduction, health and nutrition, economic growth, and labor market opportunities, as well as peacebuilding, social cohesion, and the promotion of democratic institutions. (2018, p.13).

The accounts of other actors echo this idea, linking quality education (as good learning outcomes) with increase in ‘productive employment’ (DFID, 2018, p. 9) and ‘growth and development’ (WB 2018, p. 4).

Only two international documents (Incheon declaration (2016) and a Government of Ireland 2019) in part have a different rhetoric. Both documents regard education as a human right. This implies the existence of duty bearers who are responsible for vindication of this right, while right holders are entitled to claim their right through a range of individual and collective practices. This rights-based approach to education seeks to tackle the root causes of inequalities in education.

Interestingly, regional and national documents frame their statements within a neo-liberal perspective of economic growth and human capital development rather than human rights. The Sierra Leonean Ministry of Education claims that its primary goal is: ‘to provide opportunities for children and adults to acquire knowledge and skills, as well as nurture attitudes and values that help the nation grow and prosper’ (MEST, 2018, p. i). This logic assumes that improving the country’s international competitiveness will reduce poverty.

The focus on skills acquisition to overcome poverty rather than address structural issues such as inequalities and violence embedded in the society is also reflected in the framing of an international education change agenda. Following the postulates of a scientific management paradigm, those interventions that aim to improve (measurable) learning achievement of students are prioritised, while more serious structural issues such as access to knowledge are often neglected or ignored. Although the concept of ‘safe learning environment’ understood as the absence of violence in school and gender equality is present in all documents, it is considerably less prevalent at the macro or strategic planning level. To illustrate this, we established the level of frequency of the terms ‘safe’ and ‘violence’ in the three document sets. Their frequency is greater in the micro texts (230th and 47th, respectively), followed by the meso (351st and 330th) and macro (746th and 773rd), clearly manifesting a declining presence from micro to macro.

The WB’s (2018) definition of the learning crisis does not address these issues at all, focusing instead on the various aspects of learning outcomes. Other international documents (DFID, 2018; USAID, 2018) and Sierra Leonean national educational plan (2018) address such concerns more explicitly, yet when it comes to defining desirable outcomes of education, they continue to focus on literacy and numeracy skills as the most suitable way to understand the efficiency of the educational system, another neo-liberal trope, whereby efficiency trumps efficacy (Gross & Stein, 2001). Thus, despite recognition of structural disadvantages and violence in school being problematic, positioned outside the parameters of the learning crisis there is no necessity for them to appear as part of a solution. Reframing education as acquisition of certain skills in literacy and numeracy constructs the notion of ‘quantitative effectiveness [of education] which relies on test results to verify effectiveness and quality’ (Bivens et al., 2009, p. 98), thus enabling measurement and standardised tests to dominate the education landscape.

### Reframing the learning crisis: measurement in education

Since low learning outcomes are cast as the central educational problem, their measurement becomes a central task for both international and national policy-makers. This is not a new idea and has been present in the literature even before the conceptualisation of the learning crisis. For example, a WB (Pritchett, 2001, p. 379) publication argued that ‘the quality of schooling across countries is impossible to measure without internationally comparable examination’ and declared that the WB would support the measurement of learning achievements worldwide. Measurement reinforces the conception of education as acquisition of certain skills that can (and should) be measured through national examinations, international assessments like the OECD Programme for International Student Assessment (PISA) and local-level standardised tests such as Early Grade Reading Assessment (EGRA) and Early Grade Mathematics Assessment (EGMA). This discourse of measurement was evident in all macro and meso documents, even those documents that were framed or oriented within a rights-based tradition are intent on supporting: ‘national monitoring and evaluation systems in order to generate sound evidence for policy formulation and the management of education systems as well as to ensure accountability’ (UNECSO, 2016, p. 11).

Organisations that frame their educational change work as human capital development insist that measurement is an integral part of the intervention process. USAID’s (2018, p. 6) aim ‘is to achieve sustained, measurable improvements in learning outcomes and skills development’. Similarly, the WB calls for the creation of ‘learning metrics’ that measure learning outcomes nationally and internationally, creating a ‘political space for innovation’ (2018, p. 24) and providing support for interventions that address the learning crisis. The GPE also follows this logic prioritising assessment as a major focus of its work in Sierra Leone:

The ESP [Education Sector Plan] is therefore focused on: improving the teaching and learning situation, monitoring learning through learning assessment tests and analysis of scores, improving performance in the WAEC conducted National Primary School Examination (NPSE), Basic Education Certificate Examination (BECE), and West African Senior School Certificate Examination (WASSCE). (GPE, 2018, p. 8).

National (meso) documents analysed repeat this international rhetoric, framing low scores in international standardised tests as the main evidence of low learning outcomes of students in the country, thus declaring national and regional examination results as indicators of success. Consequently, ‘measurable’ becomes synonymous with ‘valuable’ and only those skills that can be easily quantified become the fulcrum on which improved educational quality depends (Unterhalter, 2019).

### Policy rhetorics: melding macro, meso and micro

These globalised ideologies of education as acquisition of measurable skills play a significant role in the development of national educational policies especially in the context of high aid dependency in the Global South. Lingard and Ranotte (2011) argue that globalisation of educational agendas has led to the relocation of political authority beyond the nation state. This process of incorporation of global discourses in the national context is highly visible in Sierra Leone. The GPE and UNICEF (2018) developed a plan for the improvement of education there that reflects global ideas of measurement as an integral part of the reform process. This plan does not use the term ‘learning crisis’ but clearly works within this paradigm as the problem of education in Sierra Leone is framed as remarkably low learning outcomes, measured through such standardised tests as EGRA and EGMA. The plan argues that measurement of learning outcomes in numeracy and literacy is ‘central to the learning process’ (Ibid. p. 72) and sets regular measures of learning outcomes as one of the priority areas for the whole intervention plan.

National documents too are framed within the general discourse of measurement of literacy and numeracy skills as the most appropriate means of addressing the problem and monitoring progress. While international documents such as WB Report (2018), DFID (2018) and USAID (2018) discuss the problems of measurement in education in more general terms, the Education Plan developed by the Ministry of Education is more specific, repeating the statements of GPE that low EGRA and EGMA scores along with high dropout rates are the most serious problems of education in Sierra Leone. To improve this situation, measurable interventions are needed and, to capture the progress of these interventions, the Ministry suggests the use of the results of national and regional examinations as an indicator of the quality of education. Towards this end, it develops a set of numerical indicators such as ‘percentage of WASSCE candidates passing English and Maths’ and ‘percentage of learners meeting assessment criteria for their levels’ (2018, p. 57) arguing that:

The performance of candidates in school level examinations conducted by the West African Examinations Council (WAEC) provides an idea of the state and quality of teaching and learning in the schools. (2018, p. 49).

In turn, the professional standards for teachers developed by the Sierra Leone Teaching and Service Commission (2017) focus extensively on assessment practices in classrooms and the importance of exams. Arguably, in contexts where teacher education is limited, when such a climate is created, concern for and preoccupation with testing has potential to be severe, in the absence of resources and professional support. This preoccupation with testing and achievement reflects the main postulates of the scientific management paradigm and thus limits opportunities for building teachers’ pedagogical repertoires, imbued with more progressive or critical orientations. Rather, such is the pervasive presence of a testing-measurement culture in Sierra Leone ‘that quality schools and teachers are those with the best examination results’ (Wright, 2018, p. 55).

### The language of SLM: ‘grassroots’ or scientific management Chameleon?

Figure 3 has already indicated a strong presence of scientific management language in the SLM documents, particularly the literacy manual, and more surprisingly in the TLC (Teaching Learning Circles) document. While undoubtedly, these documents retain ‘bottom-up’ features, scientific management reinvents itself in different hues. For example, although assessment does not appear among the terms most associated with learning, it is prevalent nonetheless in documents pertaining to the teaching dimension of the SLM intervention. However, when the frequency of the scientific management chameleon terms (assessment, testing, measurement-EGRA) are searched for in the SLM documents, their frequency reveals a more pervasive presence.

**Fig. 5:**
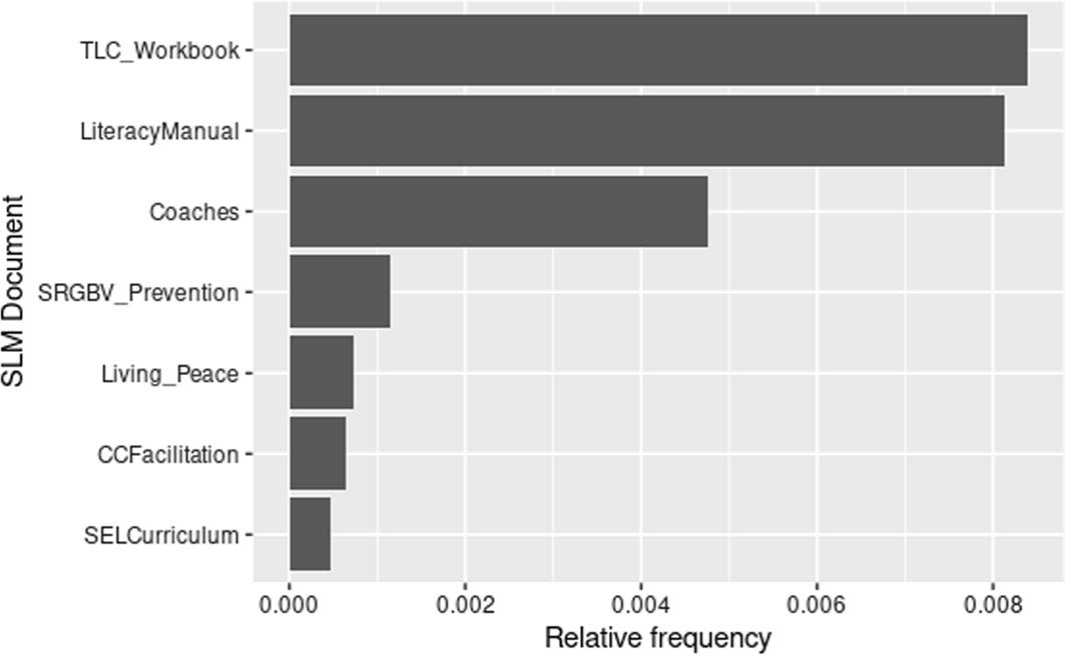
The relative frequency of ‘chameleon’ terms from the SMa paradigm in the SLM documents

Figure 5 indicates that in the three school-related documents (TLC Workbook, Literacy manual and Workbook for Coaches), the language of scientific management is significantly prevalent; much less so in relation to the community focused documents. Awareness of this degree of influence, in a context where conditions and resources are Spartan, and the knowledge base of teachers is thin or non-existent; conditions for pedagogical capacity building are far from optimal, placing a premium on coaches’ expertise, an under researched area even in much more favourable circumstances. For Concern, education is ‘one of the best routes out of poverty’ that gives ‘extremely poor children more opportunities in life and to support their overall well-being’ (Concern 2019, p. 30). Education ‘as a route’ has identifiable neo-liberal credentials; an instrumentalist education as a means of poverty alleviation and economic growth, prevalent also in the macro and meso documents. This perspective promotes a mindset or orientation that focuses on the process of skills acquisition required for poverty reduction and thus strongly influences Concern’s understanding of ‘quality education’ as good learning outcomes, free access to education along with improved school facilities (Concern 2019, p. 30), indicating considerable ‘alignment’ with quality education as articulated within the global framework of the learning crisis.

Being framed within the discourses of education as learning outcomes and their measurement, the SLM planned change is to tackle poor educational performance in Sierra Leone through addressing literacy of primary school children. However, the concept of literacy in this intervention is narrowly framed as ‘the ability to read and write’ only (Concern, 2017a), which allows Concern to measure this literacy using international standardised tests. This narrow focus on early literacy is resonant with the GPE-UNICEF (2018) assertions that literacy in first grades of primary school should be given priority in Sierra Leone due to remarkably low student EGRA scores.

Reframing education as learning outcomes that should be measured has considerable implications for teachers in their classrooms. There is widespread recognition in national and international documents that ‘business as usual’ is unacceptable, and current teaching practices need improvement. However, there is a surprising lack of discussion about the pedagogy in the classroom (see, Sayed & Moriarty, 2020). Nevertheless, an instrumentalist reframing of quality education into improvement of measurable learning outcomes funnels efforts into specific techniques that teachers can deploy to improve test scores. At that point, scientific management needs the assistance of a teacher professionalism paradigm if teacher learning through enhanced understanding and expanded pedagogical repertoires are to build their expertise.

The perception that teachers’ skills are crucial for the improvement of learning outcomes is not new and is widely accepted across the educational sector. As we have seen above, it is also present within the discourse of the learning crisis and major international actors in the educational landscape of Sierra Leone call for improvement of teaching practices in schools. The WB (2018, p. 22) advocates provision of targeted teacher training (‘often around a specific pedagogical technique’) to facilitate ‘effective teaching’. Other international documents, including Irish Aid (2018) and USAID (2018), describe their aims in terms of improvement of efficiency of teaching using specific training packages, training, and ongoing coaching, with little reference to broader pedagogical concepts.

National documents follow the same logic, focusing on specific techniques and tools intended to improve learning outcomes. Among these techniques is the development and dissemination of highly structured and detailed lesson plans to schools, arguably a version of ‘teacher proof’ curricula, anathema to teacher agency (Priestly et al., 2015). Other targeted techniques include use of instructional aids during lessons, questioning techniques and time management. Assessment techniques both formative and summative (often in a form of standardised tests such as EGRA) are presented as being integral to improving learning outcomes. It is stressed that teachers’ ability to assess students is a most important professional skill, melding measurement discourse with discourses of effective teaching. However, since the majority of teachers in the Tonkolili district are unqualified, and with modest educational attainment at best, the potential of the intervention to improve teaching and learning is hampered considerably due to the realisation that ‘differences in teacher cognitive skills can explain significant portions of the international differences in student performance’ (Hanushek et al., 2019, p. 859). In the absence of an appropriate knowledge base among teachers, teaching to the test may be the most likely outcome.

The SLM intervention is framed around the necessity to improve teachers’ skills through the focus on ‘activities [that] aim to develop the competencies related to early grade literacy of teachers’ (Concern Worldwide 2017a, p. 4). This rather narrow focus on competencies related to literacies reflects the general framing of the intervention as the improvement of the ability to read and write. In this regard, it follows the discourse of the learning crisis that focuses on the development of certain skills rather than recognising the significance of teacher agency, or building their professional capacities in an expansive manner, reflective of more progressive or transformative paradigms. Rather, SLM documents present a tool kit with basic teaching techniques. These include the use of lesson plans, letter tracing, visual aids and regular formative and summative assessment of students to improve the literacy skills of students measured through an EGRA assessment Concern Worldwide 2017a). School-level interventions are focused on teacher training and development through regular in-classroom coaching, teachers’ workshops, and support of teacher learning circles where teachers can share their experiences, along with support of school management and provision of teaching materials (Concern Worldwide 2017a; Concern Worldwide 2017b). However, in practice, the lockstep lessons plans mentioned above appear to be the pivot on which support revolves; perhaps foreshadowing the GPE assertion that ‘coaching only works if it is conceptualized, designed and implemented well’ (emphasis original) (Burns, 2020). Such designs are constrained since ‘very little [is known] about what makes for an effective coach or what a system for selecting and training an effective corps of teacher coaches should look like’, challenges particularly problematic in a context where many teachers are untrained or under-trained (Kraft & Blazar, 2017, p. 1058).

Although aspects of the SLM indicated above are evidently narrow in scope and transactional, there is evidence also of more holistic elements in the following:

Children’s educational progress will be enhanced when they live in communities where there is more support for gender equality and children’s wellbeing. (Concern Worldwide 2017a, p.2).

This assertion claims that well-being of children and gender equality in the classroom and the community may positively affect student learning outcomes. The model tries to improve learning outcomes directly through the improvement of teaching practices at the school level and indirectly through work on gender equality both in schools and in communities and supports improvement of food security and access to health services at the community level. Concern explicitly recognises community as being central to the discourse of a safe learning environment and quality education.

However, when it comes to evaluation of the SLM, literacy skills measured by standardised tests are foregrounded. This measurability of literacy skills accords with the macro discourse of measurement of learning, a perspective articulated by Concern Worldwide 2019, p. 37) when it states that it uses ‘results-based management’ in its work with the intention of finding measurable changes in the society. This core focus on measurement is also evident in the evaluation of the SLM being conducted by the authors on behalf of Concern. Though the evaluation includes a mixed-methods approach, with interviews and field observations with teachers, learners, headteachers, parents, grandparents, teenagers, and community leaders, central to the evaluation is a randomised control trial (RCT) based on EGRA scores and numeric well-being measures. As authors and researchers, we recognise that in contracting to undertake the research, we contribute to the policy process where multiple crises become a learning crisis, that is, reframed to be measurement of learning outcomes in literacy and well-being.

## Concluding discussion

The purpose of the above analysis has been to identify dominant international discourses in selected educational change policy documents, beginning with international agencies and INGOs, following through on the extent to which such policy rhetorics are ‘refracted’ (Goodson, 2004) at national level in policy texts, and the extent to which these dominant discourses chameleon-like, frame, funnel and focus the SLM planned change at the point of intervention in a rural district of Sierra Leone. Interrogated through the panoptic lens of change paradigms at our disposal, there was an absence of evidence on critical perspectives and social movement, with occasional glimpses of progressive and collaborative professionalism while the dominant international discourse quickly manifested itself, primarily inspired by prominent features of scientific management. Framing the manifold problems in Sierra Leone (and elsewhere) in this manner, from the chrysalis of scientific management the problem re-emerges as ‘learning crisis’, demanding urgent attention. Several crises are conflated into a learning crisis that is rapidly transmogrified into concern for low learning achievement, immediately necessitating a focus on learning outcomes, appropriately monitored and held to account through regular testing. While the language of crisis diminishes in its prevalence in national texts, and absent from SLM documents, the essential apparatus of measurement and testing of learning outcomes remains intact; the language of crisis has served its purpose; the chameleon lives! Thus, while it is readily acknowledged that the SLM seeks to retain and embrace a holistic approach to the multiple crises, including school and community, persistent commitment to regularly testing literacy through EGRA is entirely consistent with the tenets of scientific management, with much riding on a numeric measure of children’s well-being. This is not to predetermine the impact of the SLM model, but rather to draw attention to how such interventions are constructed whereby the architecture of international educational change discourses, even in circumstances where the language of learning crisis is dialled down considerably, the foundation of the intervention edifice has been successfully laid—regular testing of literacy, with coaching for teachers on ‘effective’ literacy skills. In the wake of No Child Left Behind Act (2001) that ratcheted up accountability and testing in its nation’s schools, while test scores increased, literacy levels declined in many instances ( Koretz, 2008, 2010; McNeil, 2000). Narrowing the focus of what counts as literacy, even in early years education, may have the undesirable consequence of improving test scores while diminishing literacy levels, perhaps simultaneously diminishing teachers’ pedagogical repertoires, particularly since they are not assessed.

Although we were not present when individuals from various international agencies and their national counterparts discussed challenges faced by the educational system in Sierra Leone, it is legitimate to ask the extent to which ‘co-construction’ (Datnow & Park, 2019) informed national policy texts? It is frequently not in the interest of politicians and policy-makers in a national arena to amplify a crisis or crises, particularly when addressing such problems requires the resources and advice of agencies that provide means towards alleviation. Ditching learning crisis while retaining the recommended treatment for the ailment—testing learning outcomes—may be perceived as a ‘legitimate compromise’ (May, 1996) on the part of policy-makers, politicians and international actors, but in the absence of an indication as to what would count as success, all concerned may be compromised while any improvement may be taken as indication of ‘success’. Although the SLM recognises the necessity to address several challenges simultaneously, and in particular support for teachers and head teachers, in addition to teenagers, communities and livelihoods, nonetheless EGRA scores and well-being measures may provide a very partial picture only of the combined efforts of schools and communities. It is also possible that the presence and contribution of international agencies allows governments to substitute interventions for more fundamental reforms such as recognition and provision of schools, reform of initial teacher education, and provision of textbooks and resources.

Co-construction, nonetheless, at the level of the school, between teachers, headteachers and coaches, has potential, but as earlier commentary has indicated, this is a sophisticated undertaking that, as yet is poorly documented. We are unaware of any input from teachers into the SLM documentation, rendering co-construction more challenging, while a degree of preoccupation with, and pervasive presence of rigid, lockstep lessons plans combined with specific teaching skills, are very suggestive of prescription rather than pedagogical capacity building, resonant with what has been described as ‘safe simulation’, a kind of ritual performance between teachers and their coaches (Hargreaves, 1994). More fundamental reform of teacher education seems a vital necessity rather than ‘tinkering towards Utopia’ (Tyack & Cuban, 1995).

Adopting a mixed methods approach to document analysis has enabled us to follow the trajectories of pivotal aspects of international educational change discourses, thus surfacing their influences on national and local texts that would otherwise remain hidden. Thus, adopting this mixed methods macro aperture to planned change allows us to restore ‘a sense of place’ to the policy-making process, recognising the ‘interface’ between the local and global, exposing ideological influences and power relations (Ball, 2006, p. 18). We note also, particularly at the macro and meso levels, a significant silence regarding pedagogical reform, confirming that ‘policies do not normally tell you what to do’ while our analysis clearly shows that these policy rhetorics ‘create circumstances in which the range of options available in deciding what to do are narrowed or changed or particular goals or outcomes are set’ (Ball, 2006, p. 21). Persisting with old recipes when faced with monumental challenges needs to be exposed, as a first step towards more transformative possibilities.

## Footnotes

1 The figures present the relative frequency of keywords. This means that the total number of words in each document/group is considered for the comparison. The relative frequency of the scientific paradigm keywords across the Macro documents is 0.005. For reference, this is at a similar level of the relative frequency of common words such as children (0.006), student (0.006), support (0.004), and work (0.004).
